# Enhanced nonlinear optical response of alkalides based on stacked Janus all-*cis*-1,2,3,4,5,6-hexafluorocyclohexane

**DOI:** 10.1016/j.heliyon.2023.e19325

**Published:** 2023-08-19

**Authors:** Muhammad Sohaib, Hasnain Sajid, Sehrish Sarfaraz, Malai Haniti Sheikh Abdul Hamid, Mazhar Amjad Gilani, Muhammad Ans, Tariq Mahmood, Shabbir Muhammad, Mohammed A. Alkhalifah, Nadeem S. Sheikh, Khurshid Ayub

**Affiliations:** a Department of Chemistry, COMSATS University Islamabad, Abbottabad Campus, Abbottabad, KPK, 22060, Pakistan; b School of Science and Technology, Nottingham Trent University, Clifton Lane, Nottingham, NG11 8NS, UK; c Chemical Sciences, Faculty of Science, Universiti Brunei Darussalam, Jalan Tungku Link, Gadong BE1410, Brunei Darussalam; d Department of Chemistry, COMSATS University Islamabad, Lahore Campus, Lahore-54600, Pakistan; e Department of Chemistry, University of Agriculture, Faisalabad, Punjab, Pakistan; f Department of Chemistry, College of Science, University of Bahrain, P. O. Box 32038, Bahrain; g Department of Chemistry, College of Science, King Khalid University, Abha, Saudi Arabia; h Department of Chemistry, College of Science, King Faisal University, Al-Ahsa 31982, Saudi Arabia

**Keywords:** Stacking, Janus molecule, NLO response, Density functional theory, Alkalides

## Abstract

Significant efforts are continuously exerted by the scientific community to explore new strategies to design materials with high nonlinear optical responses. An effective approach is to design alkalides based on Janus molecules. Herein, we present a new approach to remarkably boost the NLO response of alkalides by stacking the Janus molecules. Alkalides based on stacked Janus molecule, M-*n*-M' (where n = 2 & 3 while M and M′ are Li/Na/K) are studied for structural, energetic, electrical, and nonlinear optical properties. The thermodynamic stability of the designed complexes is confirmed by the energetic stabilities, which range between -14.07 and -28.77 kcal/mol. The alkalide character of alkali metals-doped complexes is confirmed by the NBO charge transfer and HOMO(s) densities. The HOMO densities are located on the doped alkali metal atoms, indicating their alkalide character. The absorptions in UV–Vis and near IR region confirm the deep ultraviolet transparency of the designed complexes. The maximum first static and dynamic hyperpolarizabilities of 5.13 × 10^7^ and 6.6 × 10^6^ au (at 1339 nm) confirm their high NLO response, especially for K-2-M′ complexes. The NLO response of alkalides based on stacked Janus molecules is 1–2 orders of magnitude higher than the alkalide based on Janus monomer. The high values of dc-Kerr and electric field-induced response *e.g.*, *max* ∼10^7^ and 10^8^ au, respectively have been obtained. These findings suggest that our designed complexes envision a new insight into the rational design of stable high NLO performance materials.

## Graphical abstract

**Figure:**
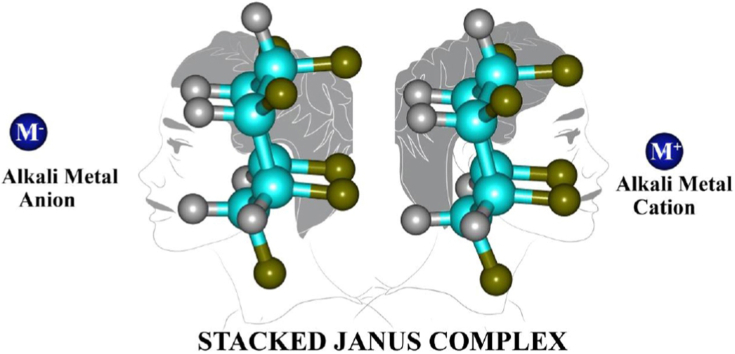

## 1 Introduction

In recent decades, the design and synthesis of materials with higher Nonlinear Optical (NLO) response, has been an area of intensive research due to applications of NLO materials in optical communication/computing, switching, dynamic image processing, and/or laser devices [1]. For this purpose, a series of inorganic materials are being utilized in NLO devices due to their asymmetric electron densities [2]. In comparison to inorganic materials, organic materials with NLO activity have gained more popularity due to their high electron delocalization [3]. Such materials exhibit small dielectric (constant) values, ultrafast response, huge laser threshold damage along with higher hyperpolarizability. Previously, notable attempts have been made to explore the nonlinear optical activity of these organic materials. Many new approaches have been developed such as bond length alternation (BLA), bond distances fluctuation, design of octupolar-molecules [3], the push-pull mechanism by introducing electron-donating or withdrawing groups [4], introducing diradical character and diffuse excess electron strategy *etc* [[5], [6], [7]].

A vast number of organic excess-electron systems with enhanced NLO responses have widely been designed by doping metals in organic molecules, including cyclic pyrroles [8], polyamines [9], fluorocarbons [6], conducting polymers [[10], [11], [12]], graphene quantum dots [13], resulting in alkalide [[14], [15], [16], [17], [18], [19]], alkaline earthide [[20], [21], [22], [23]], and electrides [5,24,25], with outstanding NLO activity. Alkalides are ionic salt containing alkali metals (such as, Li/Na/K) as anion. Alkaline earthide are complexes with negative charge on doped alkaline earth metals [26]. Electrides are a class of complexes in which electrons in space serve as anion [27,28]. These materials have much high NLO activity than those of alkalides. Normally, an alkalide is made by doping two alkali metals in organic system, where one acts as an electron source/donor and the other as an acceptor. The larger oscillator strength and small transition energies of excited states of alkali metals make alkalides better option for NLO material [29,30].

Choosing a suitable complexant is also the key to constructing alkalides with good NLO activity. *O'Hagan* et al. [31] recently synthesized a stable (facially) polarized organic molecule, called *cis*-1,2,3,4,5,6-hexafluorocyclohexane (C_6_H_6_F_6_) which exhibits outstanding properties such as remarkable dipole moment *e.g.*, 6.2 Debyes. The highest among all the aliphatic hydrocarbons [31]. C_6_H_6_F_6_ has unsymmetric electron distribution because one face is composed of hydrogen atoms while the other face contains fluorine atoms [4]. This property provides outstanding tendencies to bind with both positive and negative ions simultaneously. In addition, the alkalide nature with remarkable NLO response of C_6_H_6_F_6_ has been reported by doping with two alkali metals [7]. Sun et al. 1 theoretically reported an alkalide based C_6_H_6_F_6_ *via* doping the system with alkali metals (Li to K) as a source of electrons, where, the ns1 valence electrons of alkali metal was pushed from the fluorine side toward the hydrogen side forming an excess electron system.

In 2016, Ziegler et al. [5], synthesized the complexant of *cis*-hexafluorocyclohexane with Na^+^ and reported their remarkable anionic and cationic interaction in the gas phase. Moreover, the alkaline earthide nature of M–C_6_H_6_F_6_–M′, has also been demonstrated by Ayub and coworkers by doping with alkali metals at fluorine face and alkaline earth metal atoms on hydrogen face [29]. A step further, the NLO properties of transition metal doped C_6_H_6_F_6_ complexant have also been studied by Zhang et al. [32], and Ayub et al., [33]. Literature reveals that the stacked orientations of C_6_H_6_F_6_, such as dimer; (C_6_H_6_F_6_)_2_, and trimer; (C_6_H_6_F_6_)_3_, have been reported by Pratik et al.*,* [4] found that parallel-stacked C_6_H_6_F_6_ are most stable because of the strong hydrogen bonding (C–H⋯F). The alkaline earthide based dimer and trimer of C_6_H_6_F_6_ (M−C_6_H_6_F_6_-M′, where M is alkali metal and M' is alkaline earth metal) exhibited largest hyperpolarizability (1.5 × 10^7^ au), which was 20 fold larger than M-C_6_H_6_F_6_ (7 × 10^5^ au), This is, because, the charge density is increased significantly upon adding stacked units [32]. Therefore, the stacked system of C_6_H_6_F_6_ are the best candidates to design alkalides type nonlinear optical material. Considering the facts it is expected that the stacked dimer and trimer of all C_6_H_6_F_6_ can be used to make alkalides with remarkable nonlinear optical response [31].

Gilani and co-workers recently studied the NLO activity of a single unit of C_6_H_6_F_6_ molecule upon doping with superalkali on a fluorine site and alkaline-earth metal on H-site [6]. The authors computed NBO charge transfer, molecular orbital density, and static first hyperpolarizability of designed M_3_O-1-M′ complexes. Their reported complexes showed remarkable first static hyperpolarizability (5.2 × 10^6^ au) with a maximum NBO charge of -0.275 e^-^ on the K_3_O–C_6_H_6_F_6_–Ca complex. Similarly, Duan et al. [34], studied the NLO responses of alkaline earth metal doped (Be, Mg, Ca) complexes of Li–C_6_H_6_F_6_ monomer. The first hyperpolarizability of these designed complexes was extremely large ∼3.51 × 10^6^ au along with a sufficient negative electron density on doped metal (-0.40), computed *via* NBO analysis. Mahmood et al.*,* [35] reported the electride nature of super alkalis doped C_6_H_6_F_6_ based on the highest occupied molecular orbitals electronic densities (laid on free spaces between super alkalis and complexant). Moreover, these novel electrides exhibited high NLO activity with the first hyperpolarizability of 1.68 × 10^6^ au. Subsequently, Zhang and co-authors reported the earthide nature of AM-(C_6_H_6_F_6_)_*n*_-AEM (AM = alkali metal, AEM = alkaline earth metal and *n* = 1-3) complexes based on the shape of HOMO electron densities [32]. It was also found that the NLO activity of these complexes enhances upon increasing stacked units. The first hyperpolarizability of AM–(C_6_H_6_F_6_)_3_–AEM (AM = Li & AEM = Be) was 1.46 × 10^7^ au with an NBO charge of -0.290 e^-^ on MAE doped atom. Next, Zhang et al.*,* [36] investigated the NLO activity of AM–C_6_H_6_F_6_–MH (MH = Zn, Cd), which was remarkably high (1.0 × 10^6^ au). Another similar study was undertaken by Sun et al. [37], with Cu, Ag, and Au metals doping on AM-C_6_H_6_F_6_. In these and many other studies based on C_6_H_6_F_6_ and other related [[38], [39], [40], [41], [42]], material geometric optimization, thermodynamic stability, NBO charge densities, HOMO-LUMO orbitals, absorption studies, and static first hyperpolarizability are well-known parameters for investigating the leading applications of such materials in optoelectronic properties using DFT calculations [5,7,[33], [34], [35], [36],^43^,^44^].Other than the doped C_6_H_6_F_6_ complexes, the reported energies of HOMO, LUMO and their energy gaps of isolated C_6_H_6_F_6_ monomer are -11.19, -0.52 and 10.68 eV, respectively [6]. The UV–Vis absorption of isolated C_6_H_6_F_6_ molecule take place at 127 nm [6] Herein, we report the design of alkalide by placing alkali metal (Li, Na and K) on both the faces of the dimer (**2**) and trimer (**3**) units of C_6_H_6_F_6_ to find out the geometric stability, electronic behaviour and NLO properties of designed stacked Janus complexes within the framework of density functional theory (DFT) simulations. For this purpose, we investigated the ground state structural geometries their electronic behaviour *via* NBO charge transfer & FMO analysis, and NLO properties of newly designed complexes through static first and dynamic hyperpolarizability calculations.

## 2 Computational details

All the DFT calculations are implemented by using Gaussian09 [45] software and all the geometries and structures are visualized by using GaussView 5.0 [46]. Structures of pure stacked dimer (C_6_H_6_F_6_)_2_ and trimer (C_6_H_6_F_6_)_3_ and their dual metal doped complexes M-2-M′ (dimer) and M-3-M′ (trimer) (where, M= Li, Na and K) were optimized by using DFT functional at M06-2X/6-31+G (d, p) level of theory [47,48]. In metal-doped NLO materials, DFT has a significant role in accurately explaining their NLO activity, based on the Hartree-Fock exchange involved. Currently, long-range DFT hybrid functionals especially M06-2X is extensively been used for estimating the hyperpolarizability of NLO systems [11]. In the context of C_6_H_6_F_6_ compounds, M06-2X functional, a hybrid functional, containing 54% of HF exchange, performs exceptionally well and has been proven as a suitable DFT functional for exploring NLO properties of C_6_H_6_F_6_ [32,34,49].

The interaction energies (E_int._) of studied all-*cis* hexafluorocyclohexane complexes will be determined using equation 1:

(1) $$E_{\mathrm{int}}=E_{M-n-M\prime }\;–\;\left(E_{M}+E_{M\prime }+E_{n}\right)$$

Where *E*_*M-n-M′*_ represents the energy of dimer and trimer complexes. E_M_ and E_M′_ are the energies of alkali metals (Li, Na and K) and E_*n*_ denote the energies of (C_6_H_6_F_6_)_*n*_. Also, for the accuracy in the calculations of interaction energies, basis set superposition method is employed, that is calculated by the following equation (Eq. 2):

(2) $$E_{\mathrm{int}.CP}=E_{\mathrm{int}}+E_{BSSE}$$

where *E*_*int.CP*_ represents counterpoised interaction energy, *E*_*int*_, is the non-corrected interaction energies and *E*_*BSSE*_ is the energy of BSSE error. Vibration frequency analysis calculations are also performed on all the designed complexes in order to confirm that the designed complexes are optimized at the global minimum with no imaginary frequency.

For the evaluation of electronic properties like frontier molecular orbital (FMO) analysis, vertical ionization energies (VIE), dipole moment (*μ*), and energy gap (E_g_), M06-2X/6-31+G (d, p) level of DFT is used. Also, for NLO properties including, polarizability (*α*_0_), static first hyperpolarizability (*βo*), dynamic and second order hyperpolarizabilities, same method is used. For frequency dependent hyperpolarizabilities, we used three wavelengths i-e 1340 nm and 1906 nm. Frequency dependent hyperpolarizabilities were studied using Multiwfn code [50].

The static polarizability (***α***_**0**_**)** and first hyperpolarizability (***βo)*** *parameters were calculated by* (3), (4), *respectively.*

(3) $$\alpha_{0}=1/3\left(\alpha_{xx}+\alpha_{yy}+\alpha_{zz}\right)$$

(4) $$\beta o={\left[{\left(\beta_{xxx}+\beta_{xyy}+\beta_{xzz}\right)}^{2}+{\left(\beta_{yyy}+\beta_{yzz}+\;\beta_{yxx}\right)}^{2}+{\left(\beta_{zzz}+\beta_{zxx}+\beta_{zyy}\right)}^{2}\right]}^{1/2}$$

HOMO LUMO gaps are calculated by using equation 5.

(5) $$E_{g}=E_{L}\;–\;E_{H}$$

In above equation E_H_ denotes the energy of HOMO, while LUMO energy is shown by E_L._ Finally, the UV–Vis absorption, oscillator strength *(f*˳*)* and crucial excited state energy are computed at time-dependent (TD)-M06-2X DFT functional.

## 3 Results and discussion

### 3.1 Optimized geometries and their stabilities

Alkali metals (Li, Na, and K) doping with all possible combinations, onto both sides *e.g.,* fluorine and hydrogen of stacked dimer and trimer of C_6_H_6_F_6_ is studied. Upon doping, the total of eighteen complexes are designed, nine each with dimer (Fig. 1a) and trimer units (Fig. 1b). The geometric parameters *e.g.*, doping distances (M − F and M′-H) and interaction energies (*E*_*int*_) are computed in Table 1. The average doping distances shows that alkali metals doped more closely to the fluorine site than the hydrogen site. The M − F distances on the fluorine site range between 1.85 and 2.56 Å, while, on the hydrogen site the average M'-H distances are greater than 3.00 Å. The smallest doping distances are noticed in the case of Li metals doped complexes, which is due to their smallest atomic size. In Li-2-M′ complexes, the M′-H distances are 3.03, 3.15 and 3.63 Å, respectively. Complexes such as Na^+^-2-AM and K^+^-2-AM are also showing the similar results, like as the atomic sizes of the alkali metals increased their doping distances are also increased in similar fashion.

**Fig. 1a:**
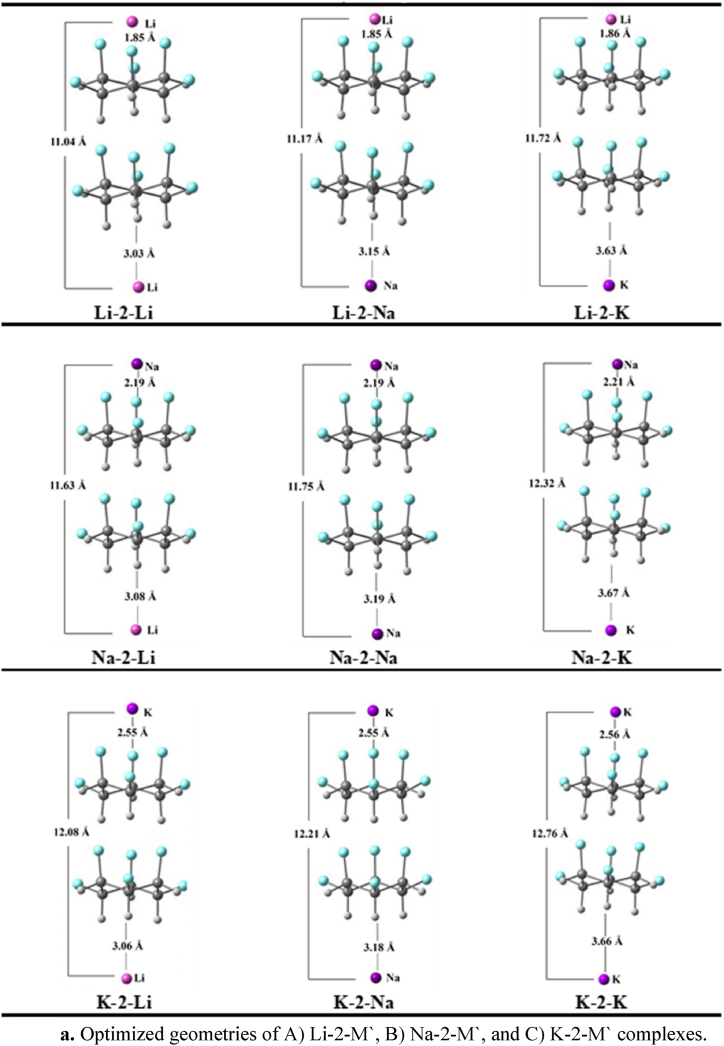
Optimized geometries of A) Li-2-M′, B) Na-2-M′, and C) K-2-M′ complexes.

**Fig. 1b:**
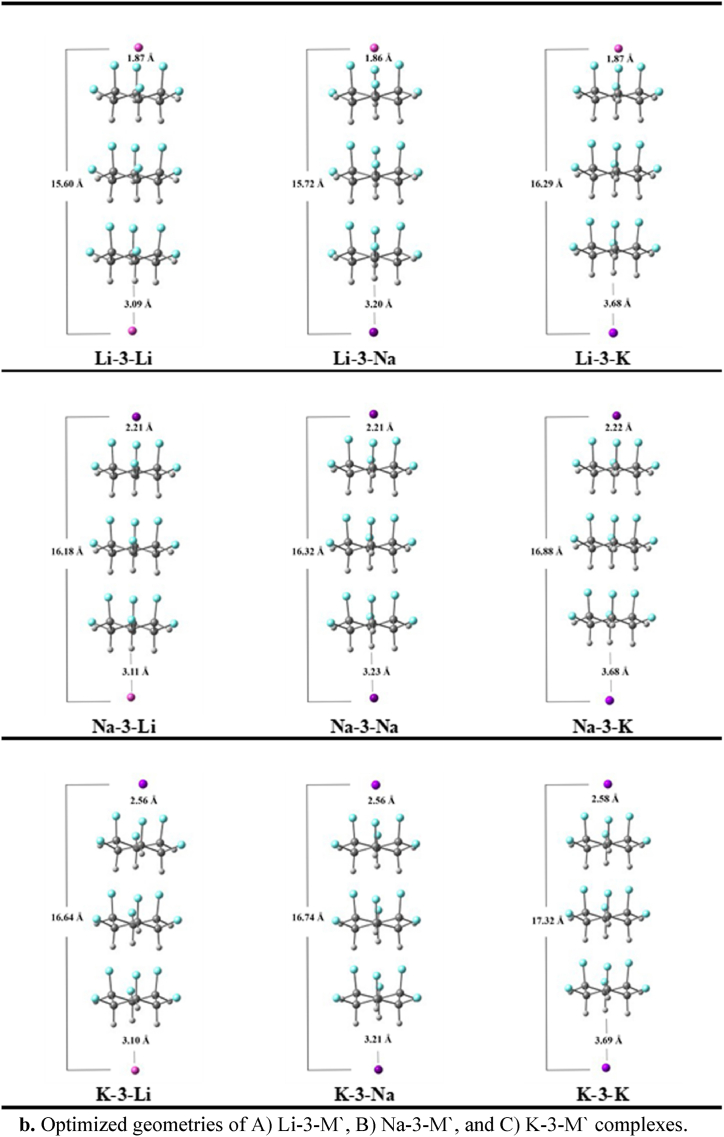
Optimized geometries of A) Li-3-M′, B) Na-3-M′, and C) K-3-M′ complexes.

**Table 1:** Geometric parameters including bond distances M-X (X = F and H), distances between doped metals at both ends (M-M′), their interaction energies (E_int_) and counterpoise corrected energies in designed M-*n*-M′ complexes.

M-*n*-M′	Symmetry	M-F Å	M'-H Å	M-M′ Å	E_int_ kcal/mol	E_int, CP_ kcal/mol
Dimer (2)						
**Li-2-Li**^**-**^	*C*_*1*_	1.85	3.03	11.04	-28.30	-27.14
**Li-2-Na**^**-**^	*C*_*1*_	1.85	3.15	11.17	-28.77	-27.63
**Li-2-K**^**-**^	*C*_*1*_	1.86	3.63	11.72	-24.89	-23.88
**Na-2-Li**	*C*_*1*_	2.19	3.08	11.63	-16.90	-15.31
**Na-2-Na**^**-**^	*C*_*1*_	2.19	3.19	11.75	-17.37	-15.83
**Na-2-K**	*C*_*1*_	2.21	3.67	12.32	-14.07	-12.62
**K-2-Li**	*C*_*1*_	2.55	3.06	12.08	-23.67	-22.90
**K-2-Na**	*C*_*1*_	2.55	3.18	12.21	-24.18	-23.49
**K-2-K**	*C*_*1*_	2.56	3.66	12.76	-20.50	-19.86
**Trimer (3)**						
**Li-3-Li**	*C*_*1*_	1.87	3.09	15.60	-28.49	---
**Li-3-Na**	*C*_*1*_	1.86	3.20	15.72	-28.87	---
**Li-3-K**	*C*_*1*_	1.87	3.67	16.29	-25.33	---
**Na-3-Li**	*C*_*1*_	2.21	3.11	16.18	-15.33	---
**Na-3-Na**	*C*_*1*_	2.21	3.23	16.32	-18.41	---
**Na-3-K**	*C*_*1*_	2.22	3.67	16.88	-17.90	---
**K-3-Li**	*C*_*1*_	2.56	3.10	16.64	-24.69	---
**K-3-Na**	*C*_*1*_	2.56	3.21	16.74	-25.20	---
**K-3-K**	*C*_*1*_	2.57	3.69	17.32	-21.84	---

Owing to the closest interaction, the Li-2-M′ complexes possessed the highest stability. For example, the interaction energies of Li-2-Li, Li-2-Na, and Li-2-K are -28.30, -28.77, and -24.89 kcal/mol, respectively. Among them, a complex containing Li at F-site and Na at H-site exhibits the highest stability. By examining the M′-H distances in K-2-M′ complexes, it can be shown that the K-doped complexes are more robust than the Li-doped complexes. Moreover, the highest charge density on K in K-2-M′ complexes is probably an important factor of their stability (*vide infra*) Other than this, the significant charge transfer (*vide infra*) in K-2-M′ complexes might be another reason for the highest stability of K^+^-2-M′ complexes. The E_int_(s) of K-2-Li, K-2-Na, and K-2-K are -23.67, -24.18, and -20.50 kcal/mol, respectively. The interaction energies for Na-2-M′ complexes, *are* -16.90, -17.37, and -14.07 kcal/mol for Na-2-Li, Na-2-Na, and Na-2-K complexes, respectively which are the least among all. Similarly, the highest BSSE corrected energies are -27.14, -27.63, and -23.88 kcal/mol for Li-2-Li, Li-2-Na, and Li-2-K, respectively (Table 1). The BSSE corrected energies for K-2-Li, K-2-Na, and K-2-K are -22.90, -23.49, and -19.86 kcal/mol, respectively. Moreover, the least values of BSSE corrected energies are for the Na series (see Table 1). Overall, the trend of the BSSE corrected energies is almost similar to that interaction energy results. Both interaction energies and BSSE corrected energies show the high thermodynamic stability of the designed complexants. In comparison between the Li, Na, and K doping on fluorine sites, the stability sequence is as follows Li-2-M′ > K-2-M′ > Na-2-M′, the following trend is in consistent with the trend reported in literature [11,51].

Quite similar to the dimer systems (M-2-M′), the alkali metal doping distances on the trimer units (M-3-M′) increase with increasing atomic radii of alkali atoms *e.g.*, Li < Na < K. For M-3-M′, the M − F and M − H interaction distances range between 1.87 to 2.57 and 3.09 to 3.69, respectively. Moreover, the interaction energy trend in M-3-M′ complexes is similar to M-2-M’ complexes. For example, the Li-doped complexes (Li-3-M′) on the fluorine site are highly stable with the interaction energy of -28.49, -28.87, and -25.33 kcal/mol, which is followed by the K-doped complexes (K-3-M′), with the energy >24.00 kcal/mol. The Na-3-M’ complexes show the least interaction energy <19.00 kcal/mol.

### 3.2 Electronic properties

#### 3.2.1 Natural bond orbital (NBO) charges

The charge transfer between doped alkali metals and (C_6_H_6_F_6_)_2_ is confirmed *via* NBO analysis. The resulting charges on the alkali atoms doped on fluorine face (Q_M-F_) and hydrogen face (Q_M′-H_) are listed in Table 2 and Table 3 for M-2-M**′** and M-3-M**′**, respectively. In the designed complexes, alkali metals on the fluoro-face bear a positive charge while those on the hydrogen face carry a negative charge. In these complexes, the charge transportation takes place based on the excess electron push and pull mechanism, where the excess electrons are pulled by fluorine atoms from doped alkali, which creates a positive charge on doped AM. Whilst, on the other side, the electrons are pushed by hydrogen, which generates a negative charge on the second doped alkali metals.

**Table 2:** NBO charge transfer at fluorine site (Q_M-F_) and hydrogen site (Q_M′-H_), vertical ionization energy (VIE), HOMO, LUMO energy & gaps (E_g_), and UV–Vis results, such as transition energies (Δ**E**), and maximum absorbance (**λ**_**max**_) of M-2-M′ complexes.

M-2-M′	Q_M′-H_ (e^-^)	Q_M-F_ (e^-^)	VIE (eV)	H_OMO_ (eV)	L_UMO_ (eV)	E_g_ (eV)	ΔE (eV)	*λ*_max_ (nm)
**(C**_**6**_**H**_**6**_**F**_**6**_**)**_**2**_	---	---	---	-10.47	-1.06	9.41	8.52	145
**Li-2-Li**	-0.671	0.940	3.54	-2.68	-1.90	0.79	1.73	713
**Li-2-Na**	-0.703	0.939	3.53	-2.69	-1.92	0.78	1.78	696
**Li-2-K**	-0.632	0.902	3.22	-2.53	-1.78	0.75	1.61	765
**Na-2-Li**	-0.629	0.89	3.42	-2.70	-2.03	0.67	1.75	706
**Na-2-Na**	-0.657	0.891	3.42	-2.71	-2.05	0.66	1.74	711
**Na-2-K**	-0.569	0.824	3.15	-2.57	-1.92	0.65	1.63	760
**K-2-Li**	-0.677	0.942	3.33	-2.53	-1.87	0.66	1.67	739
**K-2-Na**	-0.696	0.942	3.33	-2.54	-1.89	0.65	1.71	722
**K-2-K**	-0.639	0.894	3.05	-2.39	-1.75	0.63	1.54	805

**Table 3:** NBO charge transfer at fluorine site (Q_M-F_) and hydrogen site (Q_M′-H_), HOMO, LUMO energy & gaps (E_g_), of M-3-M′ complexes.

M-2-M′	Q_M′-H_ (e^-^)	Q_M-F_ (e^-^)	H_OMO_ (eV)	L_UMO_ (eV)	E_g_ (eV)
**(C**_**6**_**H**_**6**_**F**_**6**_**)**_**3**_	---	---	-10.10	-1.33	8.81
**Li-3-Li**	-0.675	0.900	-2.49	-1.98	0.50
**Li-3-Na**	-0.694	0.901	-2.50	-2.00	0.50
**Li-3-K**	-0.627	0.845	-2.38	-1.89	0.49
**Na-3-Li**	-0.608	0.849	-2.58	-2.10	0.47
**Na-3-Na**	-0.635	0.852	-2.59	-2.12	0.47
**Na-3-K**	-0.564	0.790	-2.46	-2.00	0.46
**K-3-Li**	-0.679	0.918	-2.39	-1.93	0.46
**K-3-Na**	-0.699	0.922	-2.40	-1.94	0.46
**K-3-K**	-0.62	0.848	-2.31	-1.88	0.44

Results of NBO listed in Table 2 show that there is a significant amount of charge transferred at both doping sites of designed complexes. In Li-2-M**′**, Q_M′-H_ are ranging from -0.632 to -0.703 eV, while in Na-2-M’, the Q_M′-H_ charges range between -0.569 and -0.657 eV. In K-2-M’ complexes, the Q_M′-H_ are found between -0.639 and -0.677 eV. Correspondingly, the positive charges (Q_AM-H_) of Li-series lies in the range 0.902 eV–0.940 eV, the range is 0.824 eV–0.891 eV for Na-series while for K-series it lies in the range 0.894 eV–0.942 eV. The sequence of charge transfer is like the stability trend such as Li-2-AM ≈ K-2-AM > Na-2-AM. The highest charge (negative) is transferred in the Li-2-Na^-^ complex (-0.703 eV), consistent with the highest stability of the complex. The significant net charge on alkali metals indicates their alkalide nature.

Expectedly, values of charges transferred in M-3-M**′** complexes are not significantly changed by increasing the repeating units of C_6_H_6_F_6_, however, a little decrease is observed in the negative charge density of alkali metals doped on hydrogen sites, which may affect the alkalide character of these complexes.

#### 3.2.2 Frontier molecular orbitals (FMO)

In order to further predict the electronic properties of newly designed complexes, the frontier molecular orbitals (FMOs) are examined. The HOMO, LUMO and the energy gaps (Eg) of M-2-M**′** and M-3-M′ have been calculated and given in Table 2, Table 3 and Fig. 2a,b. The E_g_ of stacked **structure-1** is 9.41 eV, however, the gaps of all the complexes are remarkably reduced below 0.80 eV. This reduction in gaps of M-2-M′ complexes is the evidence of a remarkable change in electronic properties of stacked (C_6_H_6_F_6_)_2_ upon doping with two alkali metal atoms, which is probably due to the generation of new HOMO(s) close to the Fermi energy level. The excess electrons are mainly responsible for the new HOMO(s) generation [52]. The new HOMO(s) energy levels are generated in the range of -2.53 to -2.69, -2.57 to -2.71, and -2.39 to -2.54 eV for Li-2-M′, Na-2-M′, and K-2-M′, respectively. As a result of new HOMO(s) generation, the energy gaps are significantly reduced. For example, for K-2-M′ complexes E_g_ ranges between 0.63 and 0.66 eV, followed by 0.65–0.67 eV in Na-2-M′ and 0.75–0.79 eV in Li-2-M′ complexes. Similarly, the E_g_ of bare dimer (9.41 eV) is reduced to 8.81 eV upon increasing the C_6_H_6_F_6_ repeating units to trimer (Table 3). Similarly, M-3-M′ complexes show lower E_g_ values as compared to M-3-M′ counterparts, indicating the higher charge conductivity of M-3-M′ complexes. The Eg values of Li-3-M′, Na-3-M′, and K-3-M′ complexes are 0.50, 0.47 and 0.44 eV respectively. In this study, it is found that the E_g_ of complexes is decreased with increasing atomic number of adsorbed alkali metal atoms, which is well consistent with the literature [53]. The amount of excess electrons increases as a function of atomic number, which ultimately reduces the Eg from Li to K [54,55].

**Fig. 2a:**
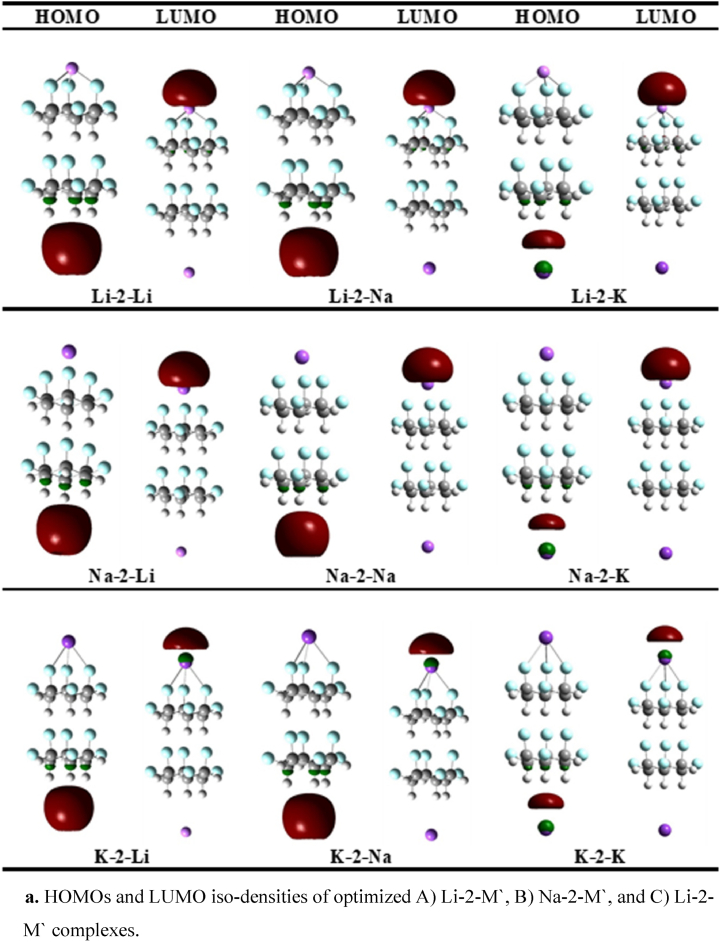
HOMOs and LUMO iso-densities of optimized A) Li-2-M′, B) Na-2-M′, and C) Li-2-M′ complexes.

**Fig. 2b:**
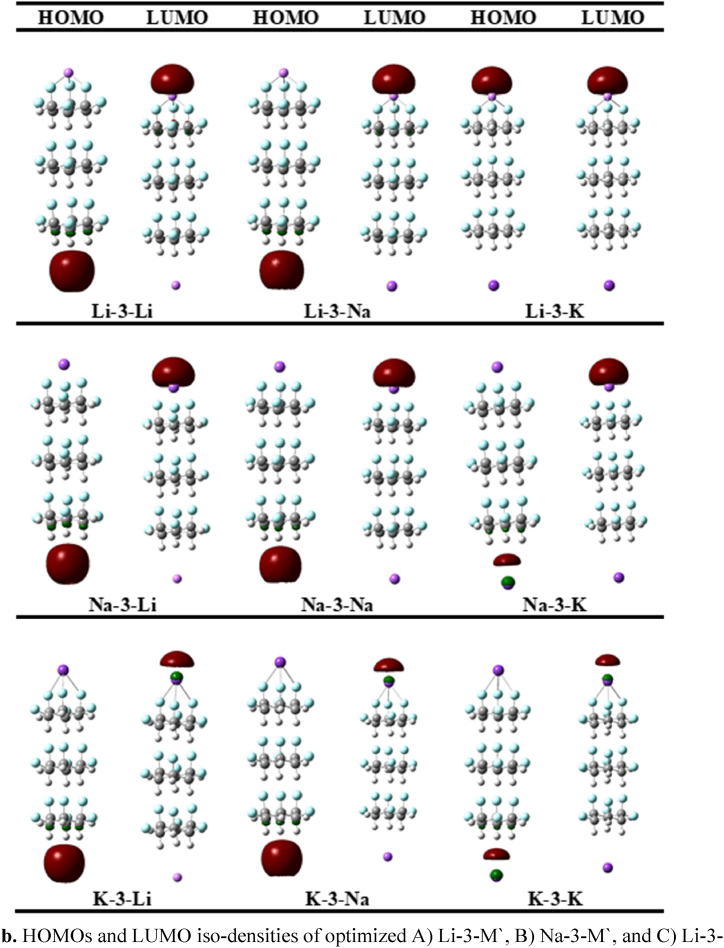
HOMOs and LUMO iso-densities of optimized A) Li-3-M′, B) Na-3-M′, and C) Li-3-M′ complexes.

#### 3.2.3 Alkalide character

**Fig. 3:**
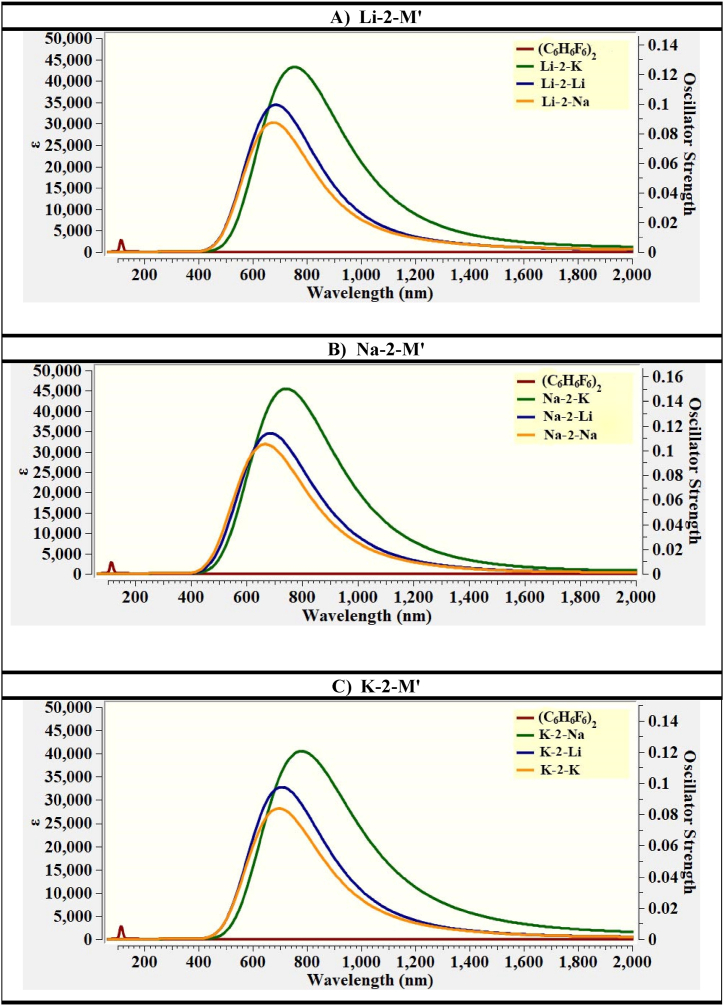
The UV–vis absorption spectra of A) Li-2-M′, B) Na-2- M′, and C) K-2- M′ complexes.

The pictorial depiction of HOMO(s) densities of M-2-M' (Fig. 2a) and M-3-M' (Fig. 2b) complexes illustrates that the HOMO densities are located on the alkali metal atoms doped on hydrogen site while the LUMO densities lie on the alkali metals doped on fluorine site, manifesting the negative and positive nature of these metal atoms on both ends, respectively. Furthermore, the HOMOs densities reside on the anionic alkali metal attributing the alkalide nature of all the designed complexes [52,[56], [57], [58]]. The alkalide character of M-*n*-M′ complexes originate because of the excess electron push-pull mechanism. Generally, in our designed complexes, the fluorine atoms of C_6_H_6_F_6_ first pull the valence one electron from the s-orbital of doped alkali metal atoms which is then pushed towards other doped alkali metal doped on the hydrogen side to create alkali metal anions.

In alkalides(s), the stability of loosely bonded electron density respectively on anionic alkali atom or in space is crucial, which has a direct relation to the vertical ionization energies (VIEs). All the designed M-2-M′ complexes acquire high VIEs *i.e.*, 3.05–3.54 eV, indicating higher electron stability [59].

#### 3.2.4 Absorption analysis

Absorption analysis has been carried out by using TD-DFT approach to investigate the laser applications of studied complexes. It is expected that NLO materials must show sufficient transparency in UV region. For this purpose, the UV–Vis spectra have been generated for pure and doped complexes. The pure C_6_H_6_F_6_ shows absorbance in UV region *i.e.*, *λ*_max_ appeared at 145 nm. After doping with alkali metals, the resultant complexes show significant transparency in UV region. The highest red shift is obtained for K-2-M′ complexes, the *λ*_max_ values for K-2-Li, K-2-Na and K-2-K are 739, 722, and 805 nm, respectively. The *λ*_max_ of K-2-M’ complexes are followed by Li-2-M’ complexes, the values range between 696 and 765 nm. The *λ*_max_ of Na containing complexes are exceptionally low. Thus, the lowest *λ*_max_ values are observed for Na-2-M’ complexes, ranging between 706 and 760 nm. Except for Na doped complexes, the UV–Vis absorption wavelength increases monotonically as the atomic number of doped alkali metals increases. The results of UV–Vis spectrums clearly indicates that the designed materials are highly efficient, due to which they can be used in many practical applications such as laser devices. So, the studied complexes can be used as an effective NLO materials. The UV–Vis absorption spectra are given in Fig. 3, while values of *λ*_max_, transition energy (ΔE) and oscillator strength (*f*˳) are displayed in Table 2.

### 3.3 NLO properties

#### 3.3.1 Static hyperpolarizability

The essential parameters regarding NLO responses are listed in Table 4. The dipole moment (***μ***_***o***_) of pure **1** is high *i.e.,* 14.84 D due to the stacking nature of the C_6_H_6_F_6_ moreover, the alkali metal doping results in a significant charge transfer which further increases the charge separation, which in-turns increases the dipole moment. Therefore, the designed M-2-M′ complexes exhibit high ***μ***_***o***_. The ***μ***_***o***_ values of Li-2-M′, Na-2-M′, and K-2-M′ complexes are ranging between 17.72 and 20.45 D, 16.21–20.25 D, and 22.87–25.70 D, respectively. For assessment of the linear response of designed complexes, the polarizabilities (***α***_***o***_) of M-2-M′ complexes are investigated. ***α***_***o***_ values of all the M-2-M′ complexes lies in the range of 694–2599 au, which is higher than to other designed complexes of C_6_H_6_F_6_ reported in the literature [60]. The values of α_o_ increase in all three series as the size of the alkali metals at the hydrogen face of 1 increase. The highest ***α***_***o***_ is observed for the K-2-K complex (2599 au), due to the soft nature of K-2-K complex. The smallest value of softness (*S*) is observed for K doped complexes, reflecting the highest reactivity of these complexes. The softness is calculated using equation 6.

(6) $$S=\frac{\eta }{2}$$

Where, H represent hardness, which can be calculated by equation 7.

(7) $$\eta =\frac{LUMO-HOMO}{2}$$

**Table 4:** NLO parameters, including the dipole moment (***μ***_***o***_), the polarizability (***α***_***o***_), the hyperpolarizability (***β***_***o***_), chemical softness (***S***), chemical hardness (***η***) variation in dipole moment (***Δμ***), oscillator strength (***f***_***o***_) and variation in excitation energy (***ΔE***) of M-2-M′ complexes.

M-2-M′	*μ*_*o*_	*α*_*o*_	*β*_*o*_	*S*	*η*	*Δμ*	*f*_*o*_	*ΔE*
**(C**_**6**_**H**_**6**_**F**_**6**_**)**_**2**_	14.84	137	478	4.71	2.35	---	---	---
**Li-2-Li**	19.62	694	1.30 × 10^6^	0.39	0.20	1.82	0.27	1.66
**Li-2-Na**	20.45	725	1.51 × 10^6^	0.39	0.19	1.95	0.25	2.68
**Li-2-K**	17.72	1379	1.00 × 10^6^	0.38	0.19	2.61	0.31	1.53
**Na-2-Li**	19.44	1257	5.17 × 10^5^	0.34	0.17	2.44	0.26	1.75
**Na-2-Na**	20.25	1289	3.91 × 10^5^	0.33	0.17	2.32	0.23	1.74
**Na-2-K**	16.21	1709	1.49 × 10^6^	0.33	0.16	2.98	0.35	1.63
**K-2-Li**	24.84	918	1.45 × 10^6^	0.33	0.17	1.72	0.24	2.62
**K-2-Na**	25.70	940	1.51 × 10^6^	0.33	0.16	1.80	0.21	2.72
**K-2-K**	22.87	2599	5.13 × 10^7^	0.32	0.16	2.48	0.28	1.45

Furthermore, the NLO responses of complexes are confirmed by computing their static first hyperpolarizability (***β***_***o***_). Overall, the ***β***_***o***_(s) of our designed (C_6_H_6_F_6_)_2_ alkalides are higher than the other similar complexes in the literature [32]. There is a non-monotonous behavior seen in the ***β***_***o***_ of M-2-M′ complexes with respect to the atomic size or atomic number of doped alkali metal atoms. The highest values of hyperpolarizability are observed for K-2-M′ complexes. The ***β***_***o***_ for K-2-Li, K-2-Na, and K-2-K complexes are 1.45 × 10^6^, 1.51 × 10^6^ and 5.13 × 10^7^ au, respectively. The ***β***_***o***_ values are decreased to 1.30 × 10^6^, 1.51 × 10^6^, and 1.00 × 10^6^ au for Li-2-Li, Li-2-Na, and Li-2-K complexes, respectively. The Na-2-M*’* complexes show least ***β***_***o***_ values, which are 5.17 × 10^5^ and 3.91 × 10^5^, 1.49 × 10^6^ au respectively for Na-2-Li, Na-2-Na, and Na-2-K. According to the literature, the ***β***_***o***_ depends inversely on VIE [61,62]. The K-2-K has the lowest VIE (3.05 eV), and thus exhibits the highest hyperpolarizability. To further analyse the factors affecting the ***β***_***o***_, two level models have been implemented [63]. The two-level model explains the inverse relation of the crucial excitation energy (***ΔE***^***3***^) and direct relation of change in *μ*_*o*_ and *f*_*o*_ with hyperpolarizability (***β***_***o***_) that can be stated as.

(8) $$\beta_{o}\propto \Delta \mu \frac{f_{o}}{{\Delta E}^{3}}$$

Based on the two-level model, the lowest value of ***ΔE***^***3***^ (1.45 eV), high values of ***f***_***o***_ (0.28), and ***Δμ*** (2.48 D) are mainly responsible for the highest ***β***_***o***_ value (∼10^7^ au) of K-2-K complex. In summary, the two-level model explains the appreciable contribution of various factors in the NLO activity of designed complexes.

**Fig. 4:**
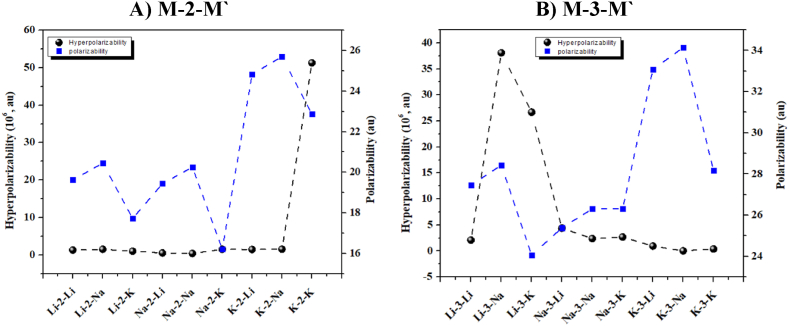
NLO performances, polarizability and hyperpolarizability values of A) M-2-M′ (dimer) and B) M-3-M′ (trimer) complexes.

In comparison, the NLO activity of designed complexes has significantly changed upon increasing the repeating C_6_H_6_F_6_ unit to trimer. For example, the Li-3-M′ complexes especially, when M' = Na and K, show a significant rise in the hyperpolarizability values. The ***β***_***o***_ values of Li-3-Na and Li-3-K complexes are 3.81 × 10^7^ and 2.67 × 10^7^ au, respectively. Moreover, these values decrease gradually on increasing number atomic number of M'-doped complexes. The non-monotonous NLO behaviour of dimer and trimer is well-comparable with those of similar complexes reported by Hou et al. 32 The polarizability and hyperpolarizability of M-2-M′ and M-3-M′ complexes are graphically compared in Fig. 4.

#### 3.3.2 Frequency-dependent/dynamic hyperpolarizability analysis

To obtain further information regarding the NLO responses of the designed alkalides, the frequency-dependent hyperpolarizability has been computed. The values including the electro-optic Pockel's effect (EOPE) and SHG of hyperpolarizability with β(−ω; ω,0) and β(−2ω; ω,ω), respectively, at two routinely used laser wavelengths (*e.g.,* 1339 and 1906 nm) are listed in Table 5. The dynamic first-order hyperpolarizability depends on the applied wavelengths. At 1339 nm, the values of EOPE range from 2.5 × 10^4^ to 6.6 × 10^6^ au and at 1906 nm these values range from 2.3 × 10^4^ to 3.7 × 105 au. The dynamic responses of the designed complexes increase with increasing applied frequencies from 1339 nm to 1906 nm with an exception in K-doped complexes. Among all the complexes, the K-2-K complex exhibits the highest EOPE value, 6.6 × 10^6^ at 1339 nm of wavelengths. The EOPE of K-2-K complex is decreased to 2.3 × 10^4^ at 1906 nm. The maximum SHG response is shown at 1339 nm *i.e.*, 6.6 × 10^6^ au. The highest SHG response is computed for K-2-K at both the wavelengths such as, 2.0 × 10^6^ and 3.8 × 10^5^ au at 1339 and 1906 au, respectively. Unlike static hyperpolarizability, the trend in dynamic hyperpolarizability is non-monotonous, however, the K-doped complexes show the highest static as well as dynamic NLO responses.

**Table 5:** Frequency-Dependent First Hyperpolarizability (β in au) at 0.0, 1339 and 1906 nm of the applied wavelengths of M-2-M′ complexes.

M-2-M′	ω=0.0 (nm)	ω =1339 (nm)		ω =1906 (nm)	
	β(0; 0,0)	β(-ω; ω,0)	β(2- ω; ω, ω)	β(-ω; ω,0)	β(2- ω; ω, ω)
**Li-2-Li**	1.3 × 10^6^	2.5 × 10^4^	8.5 × 10^5^	1.3 × 10^5^	2.5 × 10^5^
**Li-2-Na**	1.5 × 10^6^	5.3 × 10^4^	4.5 × 10^5^	1.1 × 10^5^	3.8 × 10^5^
**Li-2-K**	4.4 × 10^5^	1.4 × 10^6^	2.9 × 10^5^	3.7 × 10^5^	2.3 × 10^6^
**Na-2-Li**	6.4 × 10^5^	1.0 × 10^5^	4.6 × 10^5^	1.0 × 10^5^	1.4 × 10^5^
**Na-2-Na**	5.2 × 10^5^	6.2 × 10^4^	2.6 × 10^5^	7.3 × 10^4^	1.2 × 10^5^
**Na-2-K**	8.1 × 10^5^	2.2 × 10^5^	3.9 × 10^5^	1.7 × 10^5^	2.3 × 10^5^
**K-2-Li**	1.5 × 10^6^	5.6 × 10^4^	2.0 × 10^4^	1.0 × 10^5^	2.7 × 10^5^
**K-2-Na**	1.6 × 10^6^	5.9 × 10^4^	1.1 × 10^5^	7.6 × 10^4^	2.5 × 10^5^
**K-2-K**	1.0 × 10^7^	6.6 × 10^6^	2.0 × 10^6^	2.3 × 10^4^	3.8 × 10^5^

#### 3.3.3 Third-order nonlinear optical response

The third-order NLO responses, dc-Kerr effect γ(-ω; ω,0), and electric field-induced ESHG γ(2- ω; ω, ω), of designed alkalides, have also been calculated at, 1339 nm, and 1906 nm. The monotonous behaviors in the third-order responses of all the complexes are noticed. For example, the γ(-ω; ω,0) values are the highest in K-2-M′ complexes, followed by the Na-2-M′ complexes, such as ∼10^8^ au, whereas, ∼10^7^ au for Li-2-AM complexes at 1339 nm. Similar trends are observed at higher applied wavelengths. However, these third-order responses including γ(-ω; ω,0) and γ(2- ω; ω, ω) are significantly higher, which reveals the remarkable NLO activity of the designed alkalides (Table 6).

**Table 6:** The third-order NLO responses include, dc-Kerr effect γ(-ω; ω,0), and electric field induced ESHG γ(2- ω; ω, ω) at 0.0, 1339, and 1906 nm of the applied wavelengths of M-2-M′ complexes.

M-2-M′	ω=0.0 (nm)	ω =1339 (nm)		ω =1906 (nm)	
	γ(0; 0,0)	γ(-ω; ω,0)	γ(2- ω; ω, ω)	γ(-ω; ω,0)	γ(2- ω; ω, ω)
**Li-2-Li**	1.5 × 10^9^	7.6 × 10^7^	1.4 × 10^7^	3.3 × 10^6^	3.9 × 10^7^
**Li-2-Na**	9.6 × 10^9^	1.1 × 10^7^	7.8 × 10^8^	1.1 × 10^7^	6.1 × 10^7^
**Li-2-K**	7.3 × 10^8^	3.8 × 10^8^	1.6 × 10^9^	1.6 × 10^7^	6.4 × 10^8^
**Na-2-Li**	4.7 × 10^9^	5.3 × 10^7^	2.3 × 10^8^	3.2 × 10^7^	4.3 × 10^7^
**Na-2-Na**	4.5 × 10^9^	4.6 × 10^7^	2.5 × 10^8^	3.1 × 10^7^	3.4 × 10^7^
**Na-2-K**	1.2 × 10^8^	1.1 × 10^8^	6.2 × 10^8^	6.1 × 10^7^	4.2 × 10^8^
**K-2-Li**	7.2 × 10^9^	1.9 × 10^8^	1.0 × 10^9^	6.3 × 10^7^	1.5 × 10^8^
**K-2-Na**	7.5 × 10^9^	1.7 × 10^8^	1.9 × 10^10^	5.9 × 10^7^	1.5 × 10^8^
**K-2-K**	1.0 × 10^8^	5.1 × 10^7^	5.4 × 10^8^	6.7 × 10^7^	2.4 × 10^8^

## 4 Conclusion

Herein, the geometric, thermodynamic, electronic, NLO of alkalides based on Janus dimer (C_6_H_6_F_6_)_2_ and trimer (C_6_H_6_F_6_)_3_ with alkali metal as a source of diffuse excess electrons have been presented. These reported complexes contain significant involvement of alkali atoms as excess electron sources when doped on fluorine site, while second doped alkali atoms on hydrogen site carry negative charge by accepting these excess electrons. The interaction energies range from -14.07 to -27.63 kcal/mol for dimer complexes, whilst the energetic stability of timer complexes is increased to -28.87 kcal/mol. The small vertical ionization potentials, ranging between 3.05 and 3.54 eV, illustrate the presence of loosely bonded electrons in the designed complexes. The NBO charge analysis illustrates the negative charges on H-site doped alkali metal atoms and positive charges in F-site dopants. The HOMO densities of all the complexes reside on anionic alkali atoms, which indicate the alkalide nature of designed complexes. The UV–Vis analysis reveals that the designed complexes are transparent in deep UV-region with maximum absorption in visible and near-IR regions. The NLO responses of these complexes are investigated *via* first-static, dynamic and second order hyperpolarizability calculations. Overall, the significant NLO responses are illustrated for all the alklaides based on the generated results but the K-2-K complex shows the highest value of the first hyperpolarizability (5.13 × 10^7^ au) and β(-ω; ω,0) (2.2 × 10^6^ au) along with the third-order activity (γ_tot_) of 6.1 × 10^8^ au. However, the trend of NLO activity is completely changed to Li-doped complexant in trimer complexes. From these findings, we believe that our newly designed alkalides can be effectively used in optical and nonlinear optical devices with excellent response.

## Author contribution statement

Muhammad Sohaib: Hasnain Sajid: Sehrish Sarfaraz: Performed the experiments; Analyzed and interpreted the data; Wrote the paper.

Malai Haniti Sheikh Abdul Hamid: Tariq Mahmood: Mohammed A. Alkhalifah: Analyzed and interpreted the data; Contributed reagents, materials, analysis tools or data; Wrote the paper.

Mazhar Amjad Gilani: Muhammad Ans: Shabbir Muhammad: Performed the experiments; Contributed reagents, materials, analysis tools or data; Wrote the paper.

Nadeem S Sheikh: Khurshid Ayub: Conceived and designed the experiments; Analyzed and interpreted the data; Wrote the paper.

### Data availability statement

Data will be made available on request.

## Declaration of competing interest

The authors declare that they have no known competing financial interests or personal relationships that could have appeared to influence the work reported in this paper.
