# Supplementary material for: Enhanced nonlinear optical response of alkalides based on stacked Janus all-cis-1,2,3,4,5,6-hexafluorocyclohexane
Source: Heliyon. 2023 Aug 19;9(9):e19325. doi: 10.1016/j.heliyon.2023.e19325 (PMC10474417; doi:10.1016/j.heliyon.2023.e19325)
Supplement: Multimedia component 1 [file mmc1.docx]

**Enhanced nonlinear optical response of alkalides based on stacked Janus****all-*cis*-1,2,3,4,5,6-hexafluorocyclohexane**

Muhammad Sohaib^1^, Hasnain Sajid^2^, Sehrish Sarfaraz^1^, Malai Haniti S. A. Hamid^3^, Mazhar Amjad Gilani^4^, Muhammad Ans^5^, Tariq Mahmood^1,6^, Shabbir Muhammad^7^, Mohammed A. Alkhalifah^8,*^, Nadeem S. Sheikh^3,*^, Khurshid Ayub^1,*^

^1^Department of Chemistry, COMSATS University Islamabad, Abbottabad Campus, Abbottabad, KPK, 22060, Pakistan

^2^School of Science and Technology, Nottingham Trent University, Clifton Lane, Nottingham NG11 8NS, UK

^3^Chemical Sciences, Faculty of Science, Universiti Brunei Darussalam, Jalan Tungku Link, Gadong BE1410, Brunei Darussalam

^4^Department of Chemistry, COMSATS University Islamabad, Lahore Campus, Lahore-54600, Pakistan

^5^Department of Chemistry, University of Agriculture, Faisalabad, Punjab, Pakistan

^6^Department of Chemistry, College of Science, University of Bahrain, P. O. Box 32038, Bahrain

^7^Department of Chemistry, College of Science, King Khalid University, Abha, Saudi Arabia

^8^Department of Chemistry, College of Science, King Faisal University, Al-Ahsa 31982, Saudi Arabia

*Correspondence: [malkalifah@kfu.edu.sa](mailto:malkalifah@kfu.edu.sa) (M.A.A.-K.); [nadeem.sheikh@ubd.edu.bn](mailto:nadeem.sheikh@ubd.edu.bn) (N.S.S.) and [khurshid@cuiatd.edu.pk](mailto:khurshid@cuiatd.edu.pk) (K.A.)

**IR Spectrum:**

| **(C_6_H_6_O_6_)_2_** |
| --- |
| 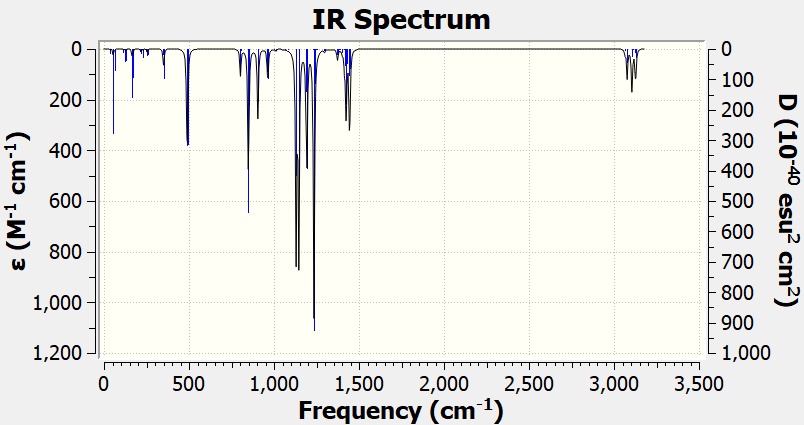 |
| **Li-2-Li** |
| **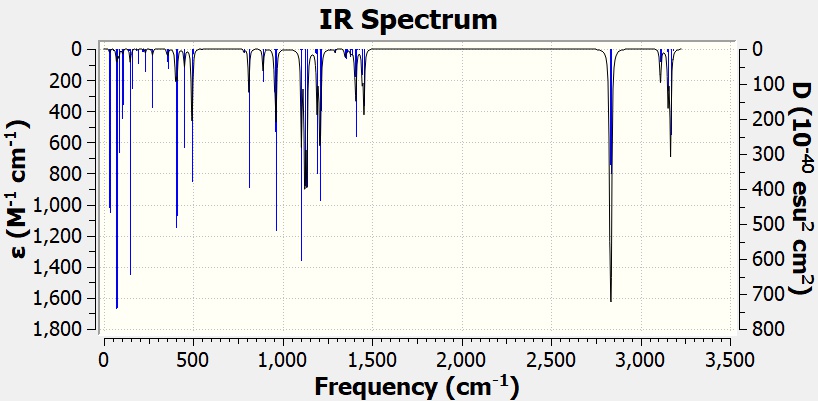** |
| **Li-2-Na** |
| **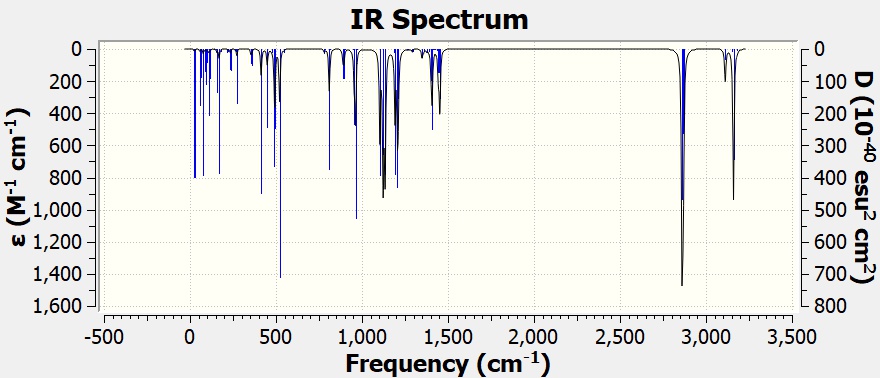** |
| **Li-2-K** |
| **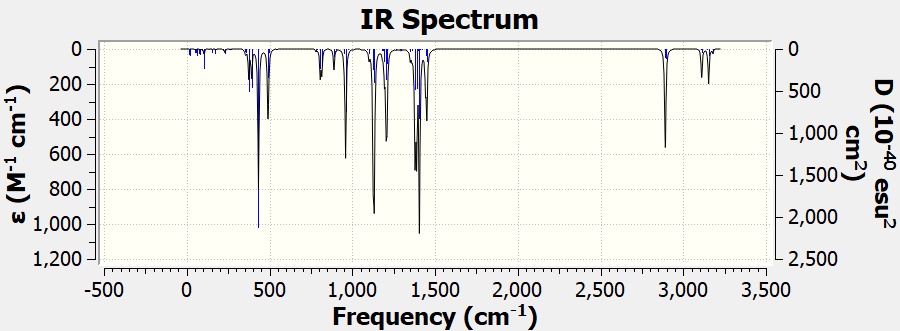** |
| **Na-2-Li** |
| **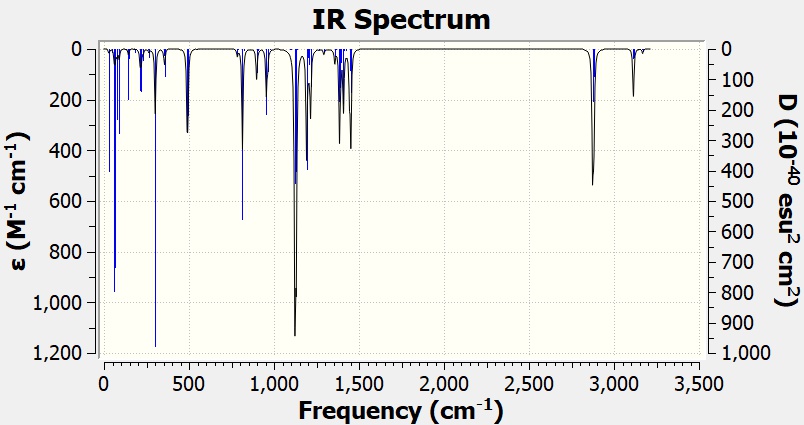** |
| **Na-2-Na** |
| **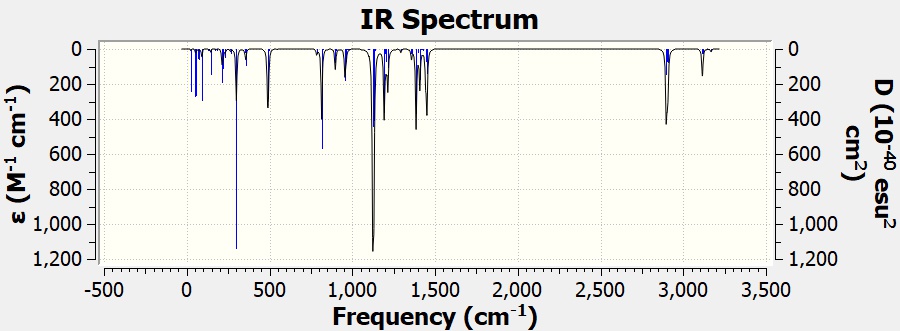** |
| **Na-2-K** |
| **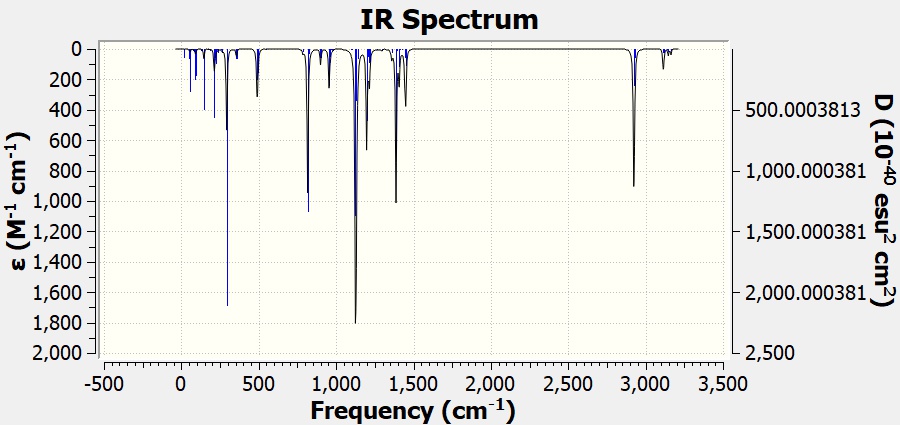** |
| **K-2-Li** |
| **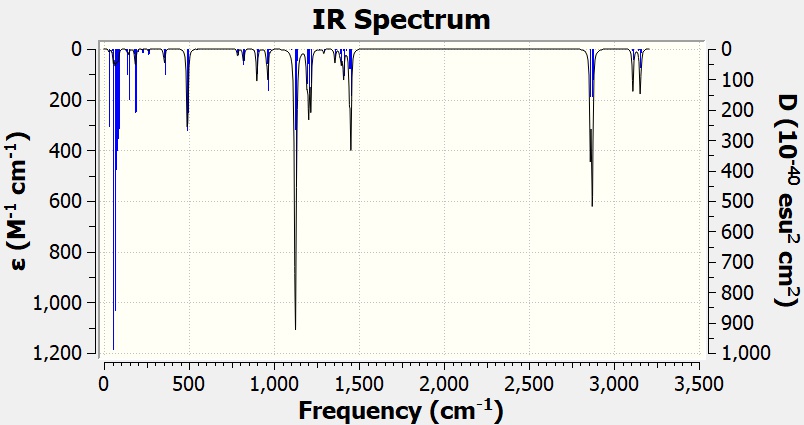** |
| **K-2-Na** |
| **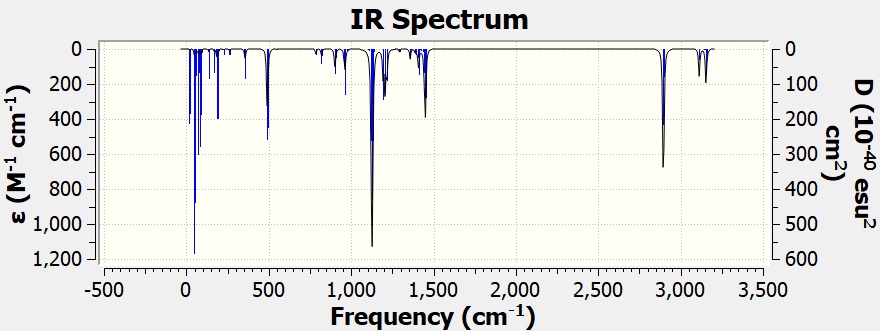** |
| **K-2-K** |
| 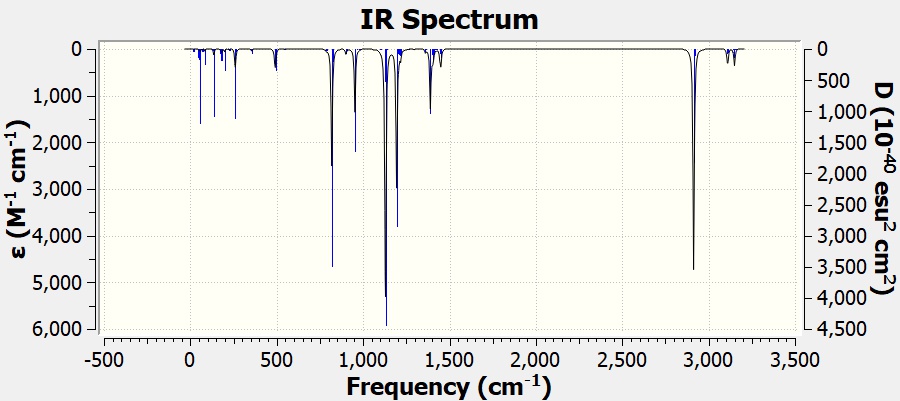 |

**Figure S1: IR spectra of 2 and M-2-M`**
